# Impact of layer count and thickness on spin wave modes in multilayer synthetic antiferromagnets

**DOI:** 10.1038/s41598-025-08393-5

**Published:** 2025-07-01

**Authors:** J. Jiménez-Bustamante, N. Vidal-Silva, A. Kákay, S. Wintz, R. A. Gallardo

**Affiliations:** 1 Departamento de Física, Universidad Técnica Federico Santa María, Avenida España 1680, Valparaíso, Chile; 2 Departamento de Ciencias Físicas, Universidad de La Frontera, Casilla 54-D, Temuco, Chile; 3 Helmholtz-Zentrum Dresden-Rossendorf, Institute of Ion Beam Physics and Materials Research, Bautzner Landstraße 400, 01328 Dresden, Germany; 4 Helmholtz-Zentrum Berlin, 12489 Berlin, Germany; 5 Departamento de Física, Universidad Técnica Federico Santa María, Avenida España 1680, Valparaíso, Chile

**Keywords:** Physics, Condensed-matter physics, Magnetic properties and materials

## Abstract

In this study, the spin-wave spectrum in multilayer synthetic antiferromagnets is calculated. The analysis focuses on the effects of varying both the thicknesses and the number of ferromagnetic layers within these structures. The results reveal that a non-reciprocal spin-wave dispersion occurs in structures with an even number of layers, while a reciprocal dispersion of two counterpropagating waves is observed for systems with an odd number of layers. As the number of layers and their thickness increase, the study identifies the distinctive presence of bulk and surface modes, with the latter being strongly affected by dynamic dipolar interactions. In multilayers with an even number of layers, such surface modes exhibit nonreciprocal behavior, maintaining their surface character only in one propagation direction. Conversely, in odd-layer systems, the symmetric counterpropagating surface modes have similar properties. Additionally, the bulk modes for both even and odd numbers of layers converge towards similar dynamic behavior as the thickness and number of layers increase. As the thickness of the ferromagnetic layers increases, the surface modes in multilayers with an odd number of layers remain localized at either the top or bottom, depending on the sign of the wave vector. In contrast, for the even case, the surface modes appear in both the top and bottom ferromagnetic layers when the layers are thin or ultrathin. However, as the ferromagnetic layer thickness increases, these modes gradually become predominantly localized at either the top or bottom of the multilayer. Finally, the study explores the application of an external magnetic field, demonstrating that surface chiral modes are absent in the saturated state, resulting in a reciprocal spin-wave dispersion. This establishes a magnetic field-mediated control over non-reciprocal localized surface modes.

## Introduction

Synthetic antiferromagnets (SAFs) are engineered magnetic materials designed to possess distinctive and highly adjustable static and dynamic magnetic properties^1,2^. Unlike natural antiferromagnetic materials, where adjacent magnetic moments inherently align oppositely, SAFs are constructed by sandwiching two or more ferromagnetic (FM) layers with a thin non-magnetic spacer layer. By precisely tuning the properties of the spacer, adjacent ferromagnetic layers can align their magnetic moments antiparallel to each other, reducing overall magnetic stray fields^1^. This antiparallel alignment is mediated by an interlayer exchange interaction, whose strength is influenced by the thickness and composition of the spacer layer^3–6^, which can comprise metallic^7,8^, semiconducting^9^, and insulating^10^ materials. Additionally, electric fields can alter the interlayer coupling, enabling transitions between ferromagnetic and antiferromagnetic states in the bilayer^11^, as experimentally demonstrated in magnetic tunnel junctions^12^. The controllable properties of SAFs can also be exploited to stabilize spin textures^2,13^. Recent studies have showcased the stabilization of antiferromagnetic skyrmions at room temperature in synthetic antiferromagnets composed of (Pt/Co/Ru) multilayers, where perpendicular magnetic anisotropy, antiferromagnetic coupling, and chiral order play a crucial role in this stabilization^14^. Besides, by increasing the total number of magnetic layers, the thermal stability of antiferromagnetic skyrmions can be favorably enhanced^14^. Consequently, such SAF structures hold promise for various applications in spintronics, magnetic storage, and sensor technologies^2,15,16^.

From a dynamic perspective, spin waves (SWs) within synthetic antiferromagnets exhibit significant nonreciprocity induced by dipolar interactions^17–23^. The antiparallel alignment breaks the system’s symmetry, causing counterpropagating waves to exhibit different wavelengths at the same frequency when traveling perpendicular to the antiparallel equilibrium magnetizations. This nonreciprocal effect is typically amplified in thick ferromagnetic films, particularly when the FM film thicknesses exceed several times the intrinsic exchange length of the magnetic material^17,22^. Recently, Girardi et al. employed time-resolved magnetic laminography to visualize the distribution of SW modes within the volume of a SAF structure, unveiling unexpected depth-dependent profiles arising from interlayer dipolar interactions^24^. They discovered intricate three-dimensional interference patterns generated by the superposition of spin waves with non-uniform amplitude profiles. The characteristics of these patterns can be manipulated by adjusting the composition and structure of the magnetic system. Magnon-magnon coupling has been observed in synthetic antiferromagnet systems under ferromagnetic resonance (FMR) conditions^25–29^. The different magnetic and geometrical properties of the coupled ferromagnetic layers inherently break symmetry, preventing the crossing between in-phase and out-of-phase resonance modes. Thus, the frequency branches exhibit hybridized phases near the anti-crossing points, resulting in an indirect gap in FMR frequencies. This coupling between in-phase and out-of-phase modes can be intensified by introducing an interlayer Dzyaloshinskii-Moriya interaction (DMI), which breaks the rotational symmetry in synthetic antiferromagnets when the DMI vector is nonorthogonal to the external magnetic field^30^.

The spin wave propagation in multilayered magnetic structures has been an essential topic in condensed matter physics, with significant advancements spanning several decades. These systems exhibit unique dynamic properties due to the interplay of dipolar and exchange interactions, leading to complex spin-wave dispersion relations and novel excitation modes. Early theoretical investigations established the fundamental behavior of spin waves in multilayered systems. For instance, long-wavelength surface spin waves in uniaxial antiferromagnets, incorporating exchange interactions within a mean-field approximation, were studied at the beginning of the 80’s^31^. The study revealed the nonreciprocity of surface modes, where spin waves propagating in opposite directions exhibit different frequencies. Shortly after, a general formula for magnetostatic spin-wave branches in finite and infinite multilayer structures was derived, demonstrating the formation of well-defined spin-wave bands with calculable edges and density of states^32^. Further advancements in the theoretical treatment of multilayers introduced new perspectives on spin-wave excitations. Authors in Ref. ^33^ identified two key features of layered structures: a spin-wave band resulting from interacting surface spin waves and a localized surface mode within the stack, emphasizing the role of dipolar interactions while omitting exchange for simplification. An effective medium approach was used later to study a semi-infinite multilayer, where the dipolar fields are averaged across film thicknesses. This macrospin approach provided a simplified yet insightful framework for analyzing superlattice dynamics^34^. The inclusion of both dipolar and exchange interactions in multilayered systems became more prominent in subsequent studies. Spin-wave calculations for various layered structures, incorporating interlayer exchange coupling, surface/interface anisotropies, and collinear magnetization orientations under an external field, were realized^35^. A new type of collective spin waves was observed, reminiscent of dipolar collective spin-wave excitations in magnetic/nonmagnetic multilayers^35^. A more rigorous theoretical framework was later formulated^36^, where Green’s function techniques were employed to integrate dipolar fields over multilayered structures, further refining the effective-medium description. Expanding on these methodologies, Ref. ^37^ developed a microscopic theory to determine surface and interface exchange interactions from spin-wave frequencies. Additionally, an entire-cell effective-medium approach was introduced, treating dipolar interactions through a macrospin perspective. The study, however, focused only on one propagation direction, limiting its exploration of counterpropagating waves. A broader treatment of magnetization dynamics in thin films and multilayers was later provided^38^, where dispersion relations for bulk and surface spin waves were derived by solving Maxwell’s equations. As reported previously^31^, the study revealed that surface modes exhibit nonreciprocity under an external magnetic field, distinguishing their behavior from bulk excitations. The role of spin-wave dynamics in revealing the static and dynamic properties of multilayers was further elaborated in Ref. ^39^. The authors analyzed spin waves in different magnetic regimes, including antiferromagnetic, spin-flop, and saturated states, establishing a clear distinction between surface and bulk modes in the FMR limit. In more recent studies, an analytical model was developed in Ref. ^40^ to describe the SW properties of synthetic antiferromagnetic composed of two coupled ferromagnetic layers with a significant thickness (several times the exchange length of the materials). The dynamic matrix method was posteriorly used to address a similar system but including bulk anisotropies^18^.

This paper investigates the spin-wave dynamics in multilayered synthetic antiferromagnets, where the ferromagnetic layers interact via dipolar and interlayer exchange interactions. The theoretical framework utilizes the dynamic matrix method^41^, an extension of the macrospin model, which allows for the treatment of ferromagnetic layer thicknesses exceeding the intrinsic exchange length of the magnetic material. Consequently, the intra- and inter-layer dipole-dipole interaction is intensified for thick ferromagnetic films, influencing the bandstructure of SWs. The study reveals that the spin-wave bandstructure is symmetric under wave-vector inversion for an odd number of layers. In contrast, the SW dispersion becomes asymmetric for an even number of layers. With increasing layers, energy gaps are opened between the bulk modes, and in-gap energy modes emerge as surface ones. This behavior resembles the topological properties of magnonic systems mediated by dipole-dipole interaction. In the case of an even number of layers, unidirectional surface modes with chiral properties emerge, being magnetic excitations localized at the top or bottom of the multilayered structure. The SW dispersions are further analyzed under the influence of an in-plane external field, which induces a transition from antiferromagnetic to ferromagnetic states in the equilibrium magnetizations. It is shown that the chiral surface modes are suppressed in the saturated equilibrium configuration.

## Theoretical model

**Fig. 1:**
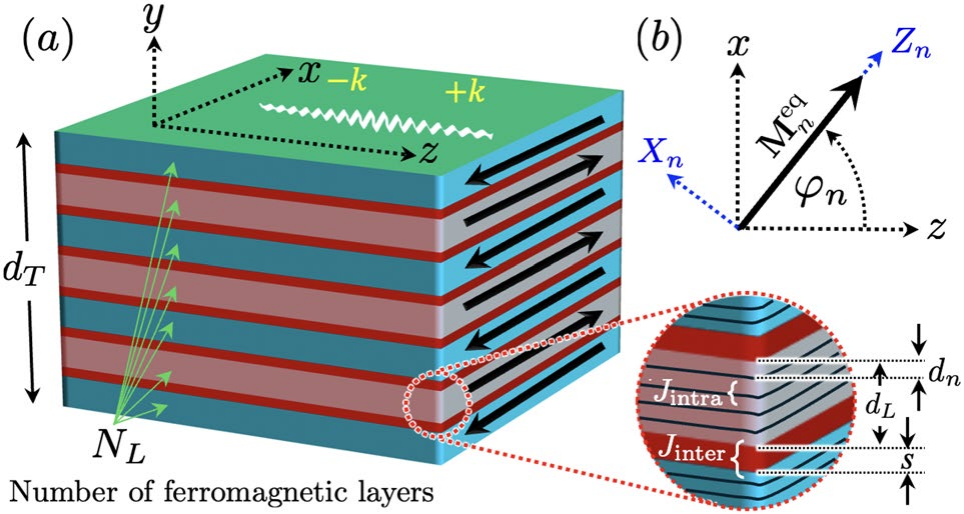
(**a**) Schematic representation of the multilayered synthetic antiferromagnet. This structure consists of $N_L$ ferromagnetic layers, each with a thickness $d_L$, separated by a spacer layer of thickness *s*. The ferromagnetic film is divided into sublayers of thickness $d_n$. The sublayers are exchange-coupled with $J_\textrm{inter}$ if they are at the interfaces in contact with the spacer and with $J_\textrm{intra}$ if they belong to the same ferromagnetic film. (**b**) Definition of the local reference system ($X_n$, $Y_n$, $Z_n$), where $Y_n = y$ is normal to the multilayer and $X_n$ lies in the film’s plane. The coordinate $Z_n$ points along the equilibrium magnetization ($\textbf{M}_{n}^\textrm{eq}$) of the *n*-th ferromagnetic layer.

The system under study is shown in Fig. 1. The multilayer structure consists of $N_L$ ferromagnetic layers with antiparallel in-plane magnetizations. Each ferromagnetic layer has a thickness denoted as $d_L$ and is separated by a spacer layer of thickness *s*. Each ferromagnetic layer is divided into multiple sublayers, each with a thickness $d_n$, and the sublayers are exchange-coupled by means $J_\textrm{inter}$ if the sublayers are at the interfaces in contact with the spacer, and $J_\textrm{intra}$ if the sublayers belong to the same ferromagnetic film. The total thickness of the structure is given by $d_T=N_L d_L+(N_L-1)s$. The spin waves are assumed to propagate along the in-plane *z*-axis, with *y* and *x* lying, respectively, in the normal and plane of the films. A local reference system ($X_n$,$Y_n$,$Z_n$) is defined, where $Y_n=y$ is normal to the multilayer and $X_n$ lies in the film’s plane. The $Z_n$ axis is aligned with the equilibrium magnetization $\textbf{M}_{n}^\textrm{eq}$ of the *n*-th ferromagnetic layer. The dynamic matrix method (DMM) is employed to obtain the spin-wave spectra in the synthetic antiferromagnet. This method divides the film into different sublayers or slabs connected through dipolar and exchange interactions^41^. The SW dispersion relation is calculated using a convergence test to ensure the correct dynamic description of a continuous film.

**Fig. 2:**
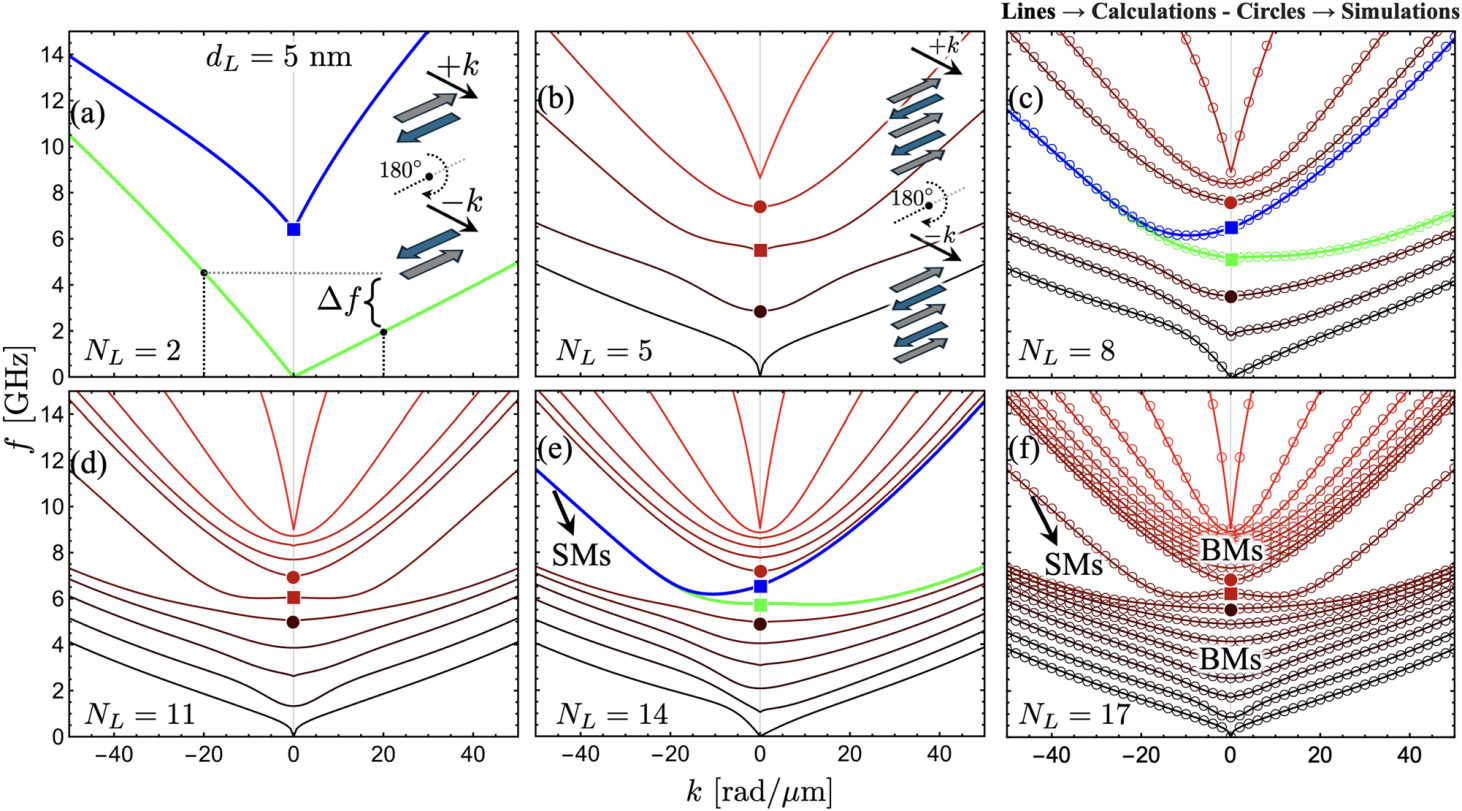
Spin wave dispersion as a function of $N_L$. The calculations are evaluated for $d_L=5$ nm and a zero external field. (**a**) illustrates the case of $N_L=2$, where the frequency shift is defined $\Delta f=\vert f(-k)-f(k)\vert$. Highlighted modes (blue and green curves) show the two most non-reciprocal modes, namely the ones with a large $\Delta f$. Bulk (BMs) and surface (SMs) modes are identified in the SW bandstructure illustrated in (**e**) and (**f**). Surface modes have chiral properties in the case of even $N_L$ [see figures (**a**), (**c**), and (**e**)], while for odd $N_L$, these modes behave reciprocally under the inversion of the wave vector [see figures (**b**), (**d**), and (**f**)]. In (**c**) and (**f**), calculated modes are compared with TetraX simulations depicted by open circles, obtaining a perfect agreement between both methods. The solid squares and circles represent highlighted modes evaluated at $k=0$, which are discussed in Fig. 3.

The time dependence of the magnetization is described by the Landau-Lifshitz (LL) equation of motion^42^, which for the *n*-th sublayer reads

1 $$\begin{aligned} \partial _t\textbf{M}_n(\textbf{r},t)= & -\gamma \mu _0\textbf{M}_n(\textbf{r},t)\times \textbf{H}_n(\textbf{r},t), \end{aligned}$$

where $\textbf{M}_n(\textbf{r},t)$ is the magnetization of sublayer *n*, $\gamma$ is the magnitude of the gyromagnetic ratio and $\textbf{H}_n(\textbf{r},t)$ is the effective field. The damping term is omitted here because it is a perturbative factor that minimally affects the spin-wave band structure. Assuming small spin deviations around the equilibrium (linearization approach), both the magnetization and the effective field can be written as $\textbf{M}_n(\textbf{r},t)=\textbf{M}_{n}^\textrm{eq}+\textbf{m}_n(\textbf{r},t)$ and $\textbf{H}_n(\textbf{r},t)=H_{Z_n}^\mathrm{{0}}\hat{Z}_n+\textbf{h}_n(\textbf{r},t)$, respectively. Here, $\textbf{M}_{n}^\textrm{eq}=M_{\textrm{s}_n}\hat{Z}_n$ corresponds to the equilibrium magnetization of the *n*-th FM film, with $M_{\textrm{s}_n}$ being the saturation magnetization of *n*-th sublayer. Also, $H_{Z_n}^\mathrm{{e0}}$ corresponds to the static $Z_n$-component of the effective field, while $\textbf{m}_n(\textbf{r},t)$ and $\textbf{h}_n(\textbf{r},t)$ are the dynamic parts of the magnetization and effective field, respectively. By assuming a harmonic time dependence and monochromatic SWs, dynamic magnetization can be expressed as $\textbf{m}_n(\textbf{r},t)=\textbf{m}_n(\textbf{r})e^{-i\omega t}$ with $\textbf{m}_n(\textbf{r})=\textbf{m}_n e^{ikz}$. Thus, the LL equation becomes

2 $$\begin{aligned} -i(\omega /\gamma \mu _0)m_{X_n}= & -m_{Y_n}H_{Z_n}^\mathrm{{0}}+M_{\textrm{s}_n} h_{Y_n}, \end{aligned}$$

3 $$\begin{aligned} -i(\omega /\gamma \mu _0)m_{Y_n}= & m_{X_n}H_{Z_n}^\mathrm{{0}}-M_{\textrm{s}_n} h_{X_n}. \end{aligned}$$

Here, $\textbf{h}_n(\textbf{r},t)$ includes the influence of all sublayers since the long-range dipolar interaction is fully taken into account. Also, it has been assumed that the collective spin waves are set to propagate along *z*, and hence, the wave vector is given by $\textbf{k} = k\hat{z}$ [refer to Fig. 1(a)]. Eqs. (2) and (3) can be written as $i\omega \textbf{m}_n=\gamma \mu _0\mathbf {\tilde{A}} \textbf{m}_n$. Once the matrix elements of $\mathbf {\tilde{A}}$ are obtained, the system can be solved as an eigenvalue problem. It should be noted that the coefficients $\textbf{m}_n$ depend on the normal direction *y*. This dependence is taken into account in the dynamic matrix method, which solves the dynamics of each sublayer located at different values of *y*. Equations (2) and (3) also contain the equilibrium condition $H_{Y_n}^\mathrm{{0}}=H_{X_n}^\mathrm{{0}}=0$. On the one side, $H_{Y_n}^\mathrm{{0}}=0$ implicates that the equilibrium magnetization is in the plane. Additionally, the in-plane equilibrium condition $H_{X_n}^\mathrm{{0}}=0$ determines the angle $\varphi _n$, which defines the ground state of each sublayer. Such a static component is given by (see Supplementary Information^43^)

4 $$\begin{aligned} H_{X_{\nu }}^\textrm{0}= H_\textrm{ext}\sin (\varphi _H-\varphi _{\nu })+\sum _{\left\langle \nu \right\rangle } \frac{J_{n \nu }\sin (\varphi _{n}-\varphi _{\nu })}{d_n \mu _{0} M_{\textrm{s}_n}}, \end{aligned}$$

where $H_\textrm{ext}$ is the external field, and $\varphi _{H_\textrm{ext}}$ is the angle that the field makes with the *z* axis. The term $J_{n\nu }$ corresponds to the exchange constant strength, and $d_n$ is the thickness of *n*-th sublayer. Note that the symbol $\left\langle \nu \right\rangle$ denotes summation over nearest neighbors, which can be written as $\sum _{\left\langle \nu \right\rangle } J_{n \nu }\sin (\varphi _{n}-\varphi _{\nu })=\sum _{\nu } J_{n \nu }\sin (\varphi _{n}-\varphi _{\nu }) \left( \delta _{n+1}^{\nu }+\delta _{n-1}^{\nu }\right)$, where $\delta _{i}^{j}$ is the Kronecker delta function ($\delta _{i}^{j}=0$ for $i\ne j$ and $\delta _{i}^{j}=1$ for $i=j$). If *n* and $\nu$ correspond to the same FM layer, the exchange term becomes $J_{n\nu }=J_\textrm{intra}$. In the case that *n* and $\nu$ correspond to the sublayers separated by the spacer *s*, then $J_{n\nu }=J_\textrm{inter}$, where $J_\textrm{inter}$ is the interlayer exchange constant [see inset in Fig. 1(a)]. Because several divisions are needed to achieve the continuous variation of magnetization along the thickness $d_L$ of the ferromagnetic layer, the results are analyzed based on a convergence criterion. Thereby, the number of divisions is truncated once the convergence of the results is reached. Besides, as the number of divisions is significant, it has been shown that $J_\textrm{inter}=2 A_\textrm{ex}/d_n$^41^, where $A_\textrm{ex}$ is the exchange constant defined in the micromagnetic approach.

Elements of matrix $\mathbf {\tilde{A}}$ for the ferromagnetic resonance case ($k=0$) were reported in Ref.^44^. In this work, such expressions are extended to a finite wave vector limit. Calculations also constitute a generalization of the ones realized in Refs.^17,18,22^, where two coupled ferromagnetic layers were considered. The matrix elements are shown in section II of the Supplementary Information^43^. In addition, part of the results are compared with numerical simulations, which are made with the open-source finite-element micromagnetic package TetraX ^45^. The propagating-wave dynamics-matrix approach, based on the linearized Landau-Lifshitz equation in the vicinity of a stable equilibrium state, implemented in this software directly yields the eigenfrequencies and the eigenvectors (mode profiles), allowing for a fast calculation of the spin-wave spectrum even for a multilayer system with a large number of layers, without the need for post processing^46^. In TetraX, the dipolar field is obtained from the magnetic potential, which is calculated using the hybrid finite-element and boundary-element method for propagating waves developed in Refs.^46,47^.

## Results and discussion

The following parameters are used for the calculations: saturation magnetization $M_\mathrm{{s}} =800$ kA/m, exchange constant $A_\mathrm{{ex}}=10$ pJ/m (exchange length $\ell _\mathrm{{ex}}=4.46$ nm), and spacer thickness $s=0.3$ nm. The interlayer exchange constant is $J_\textrm{inter}=-0.1$ mJ/m$^2$. Convergence of the modes is achieved with 20 divisions in each FM layer for all cases.

Figure 2 shows the SW dispersion for a different number of layers ($N_L$) and a fixed thickness of each FM film ($d_L=5$ nm). The case depicted in Fig. 2(a) ($N_L=2$) corresponds to the typical synthetic antiferromagnet composed of two FM layers. The nonreciprocity obtained in the SW dispersion is consistent with the results observed in Ref. ^17^, where both optic and acoustic modes have an asymmetric SW dispersion. This nonreciprocity arises from dipolar interactions, as the dynamic dipolar energy is reduced exclusively in one propagation direction, where the stray fields and dynamic magnetizations establish a flux-closure dynamic state^17^. The case with $N_L=5$ shown in Fig. 2(b) illustrates a reciprocal bandstructure where the number of modes is the same as $N_L$ due to the degree of freedom of the system. Such reciprocity in the bandstructure characterizes the cases with an odd number of layers, as shown in Fig. 2(d) and (f). In contrast, in the cases with an even number of layers, the SW dispersion is always asymmetric, as seen in Fig. 2(a), (c) and (e). The observed reciprocity in systems with odd $N_L$ and nonreciprocity in those with even $N_L$ can be intuitively understood through a simple picture that involves a 180$^{\circ }$ rotation around the equilibrium magnetization axis. As illustrated by the inset in Fig. 2(a), when considering propagation towards the $-z$ direction (or $-k$ in the SW dispersion), this is equivalent to rotating the system by 180$^{\circ }$ and then allowing wave propagation in the original positive wave vector direction. For systems with even $N_L$, this rotation results in a configuration where the propagation characteristics for $-k$ and $+k$ differ, indicating nonreciprocity as shown in Fig. 2(a). Consequently, as depicted in the inset of Fig. 2(b), for systems with odd $N_L$, the 180$^{\circ }$ rotated system is identical to the unrotated system. Therefore, the propagation for positive and negative wave vectors remains unchanged, leading to the reciprocal behavior of the SWs. This symmetry under rotation highlights the fundamental difference between systems with even and odd numbers of layers, explaining the observed propagation characteristics.

**Fig. 3:**
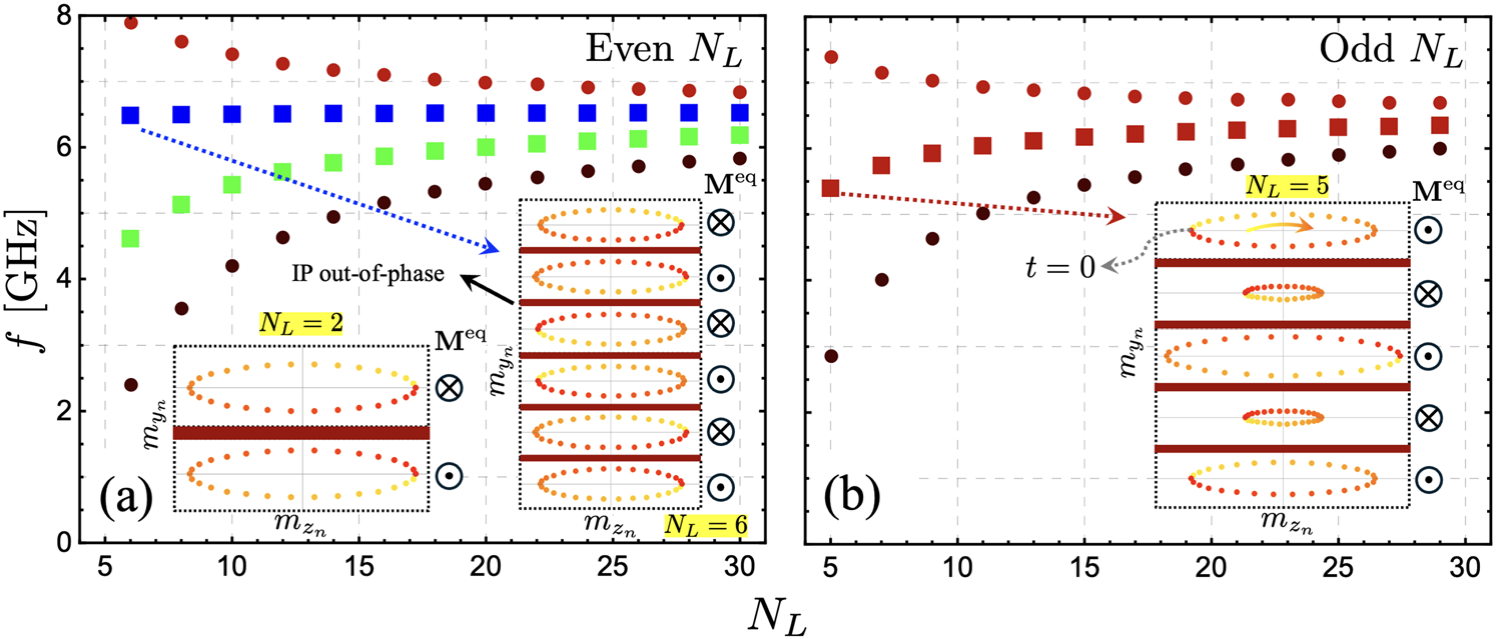
Surface modes (squares) and the modes around them (filled circles) are shown as a function of $N_L$. The limit $k=0$ is considered in the evaluation for the cases of even (**a**) and odd (**b**) $N_L$. In (**a**), the insets illustrate the magnetization oscillations for the cases $N_L=2$ and $N_L=6$. In (**b**), the inset shows the magnetization oscillations evaluated at $N_L=5$. The time goes from zero to the period as the color goes from yellow to red, respectively.

As the number of FM layers increases (for even $N_L$), the low- and high-frequency modes tend to have $\Delta f \rightarrow 0$, while two modes, highlighted with blue and green colors, have a notable nonreciprocity. For a large $N_L$, the low- and high-frequency modes (in the calculated frequency range) behave similarly for even and odd numbers of layers, evidencing a tendency toward a symmetrical SW dispersion, as depicted in Fig. 2(e) and (f). Such modes with high and low frequencies are concentrated into two densely populated branches, which are best appreciated for a large $N_L$. Due to the significant excitation observed throughout the entire thickness (see Fig. 5 for further details) at a finite wave vector, these branches will be referred to as bulk modes (BMs)^39,48–50^. An energy gap appears between the BMs, and in-gap surface modes (SMs) can be identified^39,48^. These modes have a completely different nature depending on whether $N_L$ is even or odd. If $N_L$ is even, the SMs behave nonreciprocally, as shown in Fig. 2(e). In this case, and for a large value of $N_L$, the SMs behave as bulk modes for positive wave vectors ($k>0$), while for $k<0$ they behave as genuine surface modes, as discussed below. If $N_L$ is odd, as depicted in Fig. 2(f), the SMs describe a symmetric dispersion, having the same dynamical energy for $\pm k$.

From Fig. 2, it is observed that for even $N_L$, the surface modes correspond to two distinct modes, whereas for odd $N_L$, only a single surface mode is present. As $\vert k\vert$ increases, the two modes for the even $N_L$ case become increasingly degenerate, eventually decoupling for short wavelengths. The frequencies of SMs are similar for both even and odd $N_L$, but only for negative wave vectors. However, for $k\ge 0$, their behavior differs. Fig. 3 illustrates the SMs (squares) and the surrounding modes (filled circles) evaluated at $k=0$ as a function of $N_L$. One particularly interesting feature is the frequency of the blue surface mode in Fig. 3(a). This mode (blue square) remains nearly independent of the number of layers, whereas the other modes exhibit a clear dependence on $N_L$. Still, for $N_L=2$, its frequency remains constant at approximately 6.5 GHz, as shown in Fig. 2(a) (blue square). This behavior can be understood by analyzing the dynamic magnetization profiles, as shown in the insets of Fig. 3(a). In the case of $N_L=6$, the magnetization oscillations within each coupled layer exhibit an in-plane (IP) out-of-phase oscillation. Given that the IP dynamic magnetization component is dominant, the interlayer exchange coupling has little influence on these oscillations, causing the coupled layers to oscillate almost independently. As a result, their dynamical energy remains similar to that of two antiferromagnetically coupled layers [see the inset of Fig. 3(a)]. Naturally, as interlayer exchange coupling increases, the modes become more coupled, leading to variations in the blue square-labeled mode as $N_L$ increases. For instance, if the interlayer exchange is increased by an order of magnitude ($J_\textrm{inter}=-1$ mJ/m$^2$), the frequency variation of this mode is only about 1 GHz between $N_L=6$ and $N_L=30$ (not shown), which remains relatively small. In contrast, the SM behavior for the odd $N_L$ case follows a different trend, as illustrated in Fig. 3(b). The inset of this figure shows that for $N_L=5$, only the central layer exhibits an IP out-of-phase oscillation with the rest of the layers, making this SM notoriously dependent on $N_L$. Note that the mode profiles shown in the insets of Fig. 3 do not exhibit surface characteristics because they are evaluated at $k=0$, where no dynamical dipolar interaction occurs between the FM layers. The surface nature becomes evident at finite wave vectors, where, due to the dipolar interaction, magnetization oscillations become more concentrated at the surfaces of the multilayer system.

**Fig. 4:**
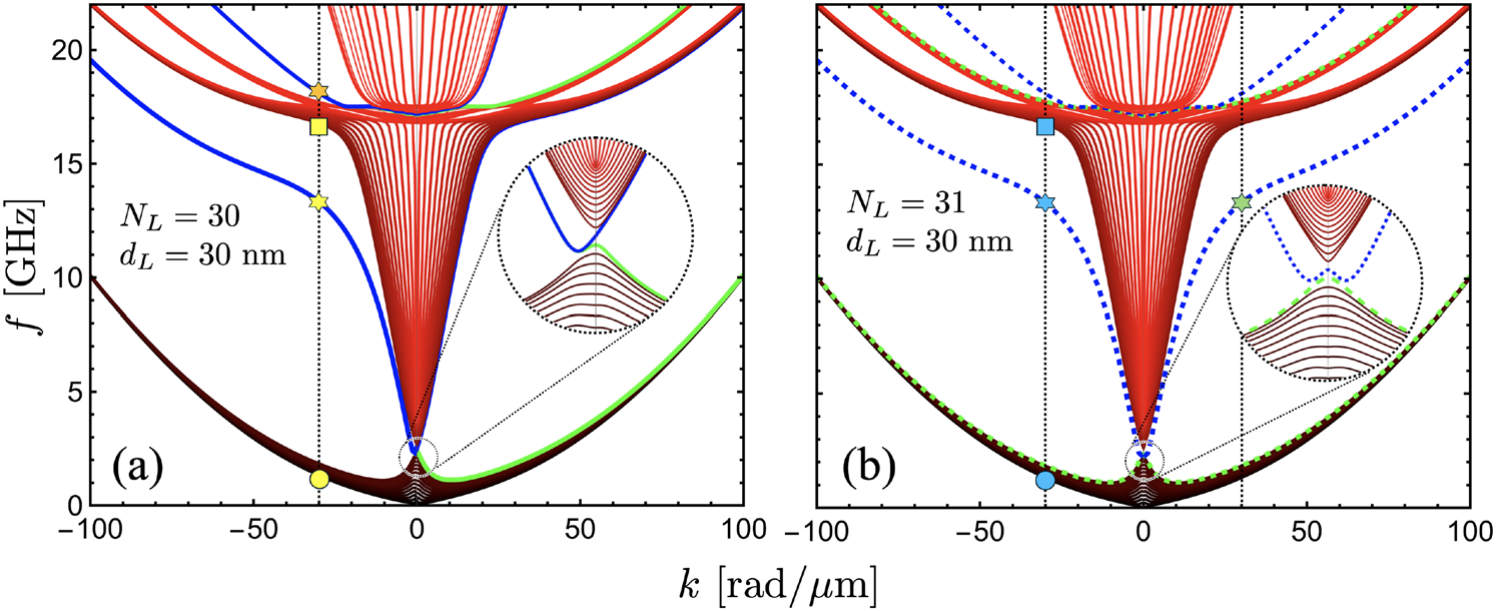
Spin-wave dispersion for $d_L = 30$ nm. In (**a**), the case with $N_L = 30$ is illustrated, while in (**b**), the number of layers is $N_L = 31$. Bulk modes are shown on a color scale from black to red, and the surface modes are highlighted in blue and green. Note that some of the surface modes can acquire bulk properties depending on the parity of $N_L$ and the sign of the wave vector. The insets in (a) and (b) provide a magnified view of the modes within a small wave vector range, where the peculiar behavior of the surface modes can be appreciated. Circles, squares, and stars highlight the dynamic states evaluated at $\vert k\vert =30$ rad/$\mu$m, which are discussed in Fig. 5.

**Fig. 5:**
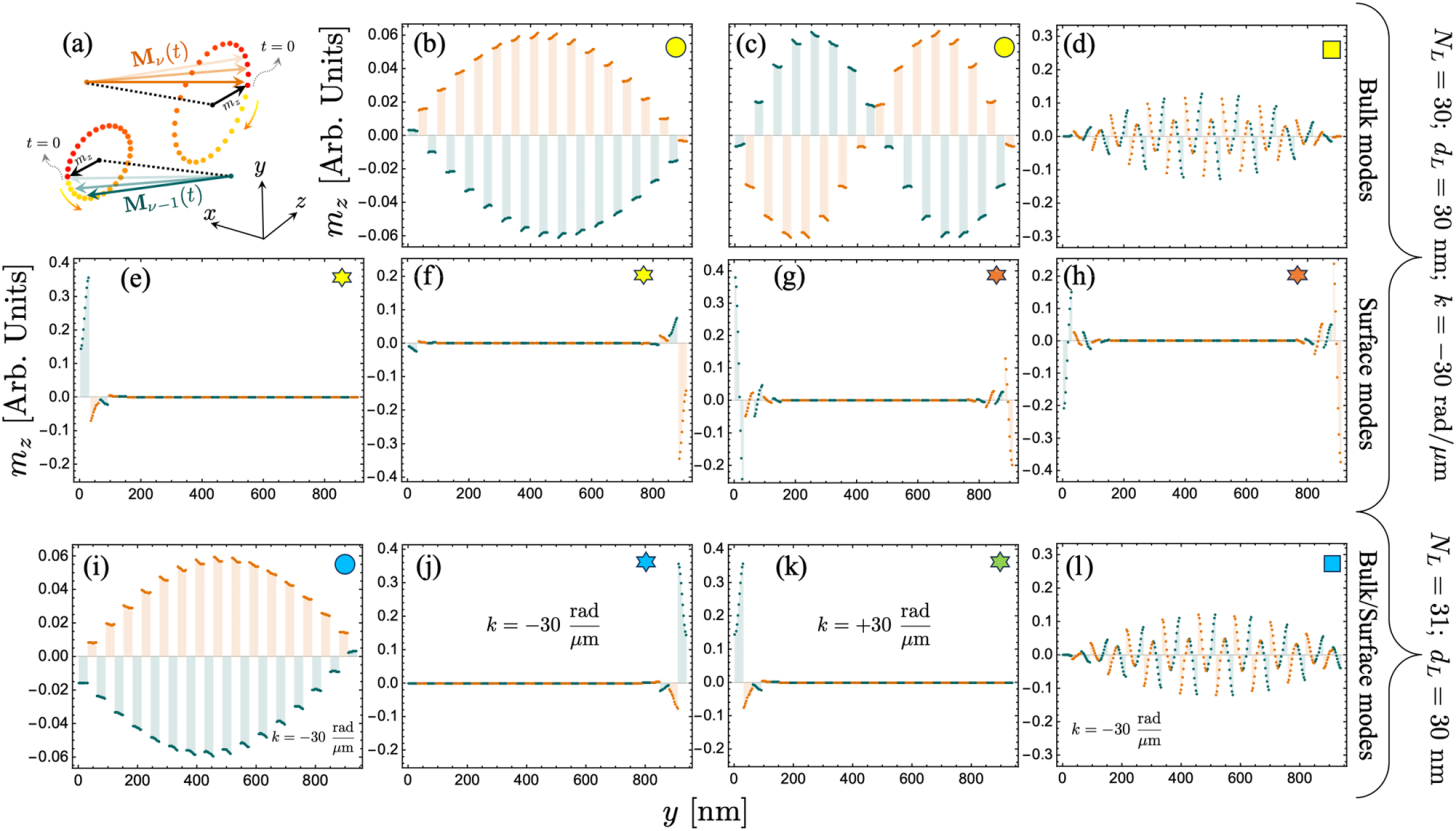
In-plane dynamic magnetization component $m_z$ as a function of the normal coordinate *y*. Calculations are realized for the bulk and surface modes highlighted by markers (circles, squares, and stars) in Fig. 4. In (**a**), a schematic representation of the magnetization oscillations is depicted. In (**b**–**h**), the case with $N_L=30$, $d_L=30$, and $k=-30$ rad/$\mu$m is shown, where the bulk (**b–d**) and surface (**e–h**) modes are plotted as a function of the multilayer thickness. In (**i–l**), the system with $N_L=31$ and $d_L=30$ is considered. Panels (**i**) and (**l**) show the bulk modes, evaluated at $k=-30$ rad/$\mu$m, against the thickness of the structure. In (**j**) and (**k**), the low-frequency surface waves are depicted for $k=-30$ rad/$\mu$m and $k=+30$ rad/$\mu$m, respectively.

In order to enhance the influence of the dynamic dipolar interaction, multilayered SAFs composed of $N_L=$ 30 and 31 are considered, where the FM thickness of each layer is $d_L=30$ nm. Fig. 4(a) shows the case $N_L=$ 30, where the inset magnifies the behavior of the surface modes at small wave vectors. In this dispersion, the low-frequency bulk modes become concentrated within a narrow frequency range. This behavior is particularly pronounced at large wave vectors, where these bulk modes tend to have the same frequency. This behavior can be anticipated since the mode quantization due to the confinement along the thickness becomes irrelevant for a large thickness of the multilayer system. Indeed, in a standard physical picture, the perpendicular standing modes with open boundary conditions exhibit different frequencies due to a term of the form $k_{\perp }=n\pi / d$^51^, where *n* is an integer number and *d* is the film thickness. Thus, as *d* increases, the frequencies for different values of *n* tend to be similar. In contrast, the surface modes are still clearly identified in the bandstructure. For the case shown in Fig. 4(a), the chiral behavior is enhanced with the increase of the multilayer thickness. Additionally, higher-order SMs are also present for a high-frequency range, evidencing a similar behavior reported in other systems^52–54^. In the case $N_L=$ 31 [Fig. 4(b)], the surface modes are also present, but in this case have reciprocal properties. By comparing Fig. 2(a) and (b), one can observe that a similar SW spectrum is obtained for $N_L=$ 30 and 31 at negative wave vector ($k<0$), while for $+k$, only bulk modes are present for $N_L=$ 30.

To illustrate the surface and bulk characteristics of the modes, spin-wave profiles are calculated for a fixed wave vector. In Fig. 5(a), an illustration of two antiparallel magnetizations is shown, where the equilibrium magnetization component points along $\pm x$, and $m_z$ ($m_y$) represents the in-plane (out-of-plane) dynamic magnetization component. Fig. 5(b–h) show selected modes evaluated at $k=-30$ rad/$\mu$m, $N_L = 30$, and $d_L=30$ nm (see geometric markers in Fig. 4), as a function of the normal distance *y*. The in-plane component $m_z$ of the two lowest frequency modes is depicted in Fig. 5(b) and (c) [see circle in Fig. 4(a)]. In these calculations, a small variation of the dynamic magnetization inside each ferromagnetic layer is observed, while the overall modulation of the magnetization along the entire structure follows the typical quantization structure^55^. Specifically, the low-frequency mode tends to be uniform, while the second mode has a node at the center of the multilayer. Due to the significant thickness of the multilayer $d_T=N_L d_L+(N_L-1)s=908.7$ nm, the dynamical energy, or frequency, of these two bulk modes is quite similar, as shown in Fig. 4(a) (see yellow circle), where the first fourteen low-frequency modes have a similar frequency at $k=-30$ rad/$\mu$m. In Fig. 5(d), the seventeenth mode [see yellow square marker in Fig. 4(a)] is plotted. This mode, together with the next thirteen modes, corresponds to those with one node inside each ferromagnetic layer. This is observed in Fig. 5(d), where within each FM film, the magnetization component $m_z$ has negative and positive values. Because such modes are excited throughout the entire thickness of the system, they are also referred to as bulk modes.

Regarding the surface modes for $N_L = 30$, there are no such modes for positive wave vectors, as shown in Fig. 4(a). Therefore, the low- and high-frequency surface modes are evaluated for the negative wave vector $k=-30$ rad/$\mu$m. Fig. 5(e-f) shows the low-frequency surface modes, which are degenerate in frequency, as shown in Fig. 4(a) (see yellow star). As observed in Fig. 5(e-f), these modes are strongly localized at the top or bottom of the nanosystem with similar dynamic magnetization profiles. It is noted that the dynamic magnetization inside each FM layer changes along the normal direction but always with an in-phase oscillation. For the high-frequency surface mode [see orange star in Fig. 4(a)], similar properties of the surface modes are obtained, as illustrated in Fig. 5(g-h). Nonetheless, the magnetic oscillations within each ferromagnetic layer are now out-of-phase, with a node close to the center of the FM film. Therefore, with increasing frequency, additional surface modes are expected to appear following the standard node distribution. For the positive wave vectors, as mentioned before, such surface modes do not exist. Specifically, these modes exhibit a unidirectional nature, which is a relevant property from both a fundamental and practical point of view.

**Fig. 6:**
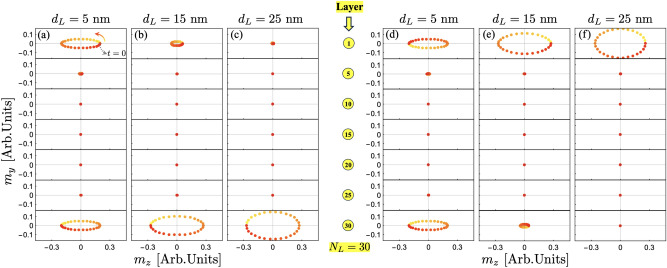
Spin wave profiles of the surface modes for the case $N_L=30$. (**a–c**) corresponds to the surface mode highlighted by blue in Fig. 4, while (**d–f**) corresponds to the green one. The dynamic magnetization components are evaluated at $k=-30$ rad/$\mu$m, while the thickness of the ferromagnetic layers are $d_L=5$ nm, $d_L=15$ nm and $d_L=25$ nm. For better visibility, the SW profiles for ferromagnetic layers 1, 5, 10, 15, 20, 25 and 30 are illustrated. The time goes from zero ($t=0$) to the period as the color goes from yellow to red, respectively.

**Fig. 7:**
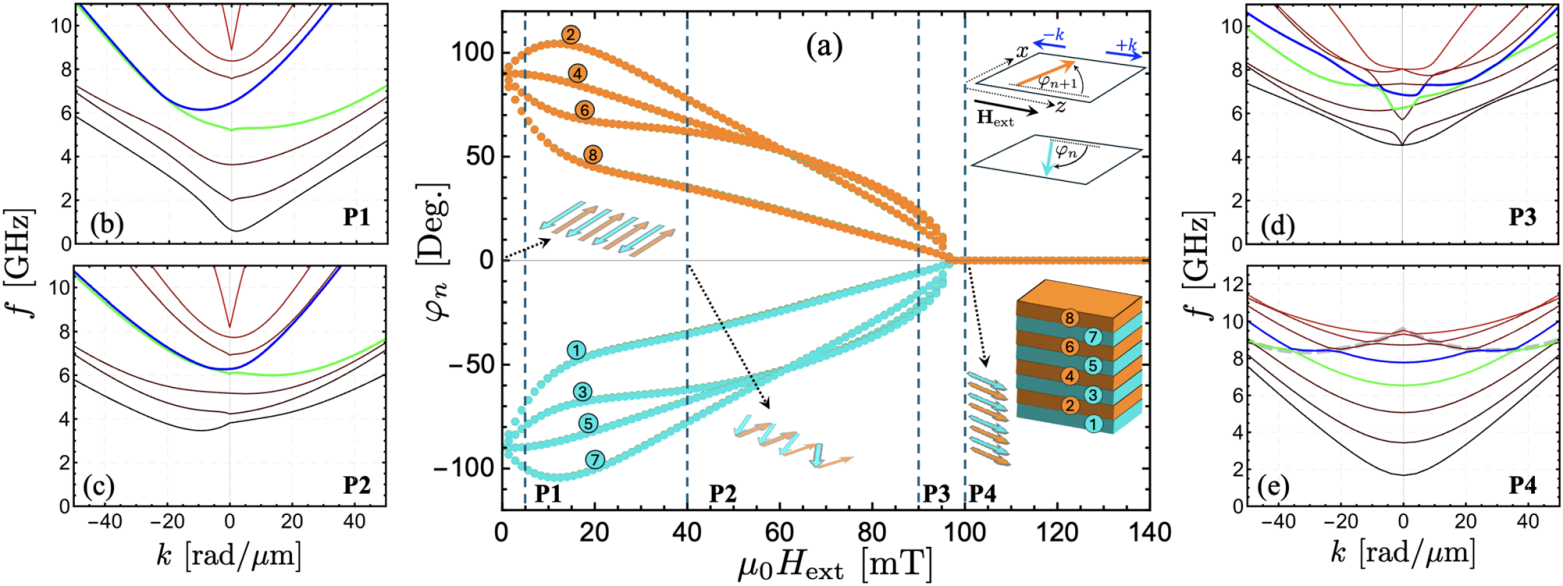
(**a**) equilibrium in-plane angle $\varphi$ as a function of the external field, which is applied along the *z* axis. The angle $\varphi$ is measured from the *z* axis, so that $\varphi =-90^{\circ }$ ($\varphi =90^{\circ }$) means that the magnetization is oriented along the $-x$ (*x*) axis. Points P$_1$ ($\mu _0H_\textrm{ext}= 5$ mT), P$_2$ ($\mu _0H_\textrm{ext}= 40$ mT), P$_3$ ($\mu _0H_\textrm{ext}= 90$ mT), and P$_4$ ($\mu _0H_\textrm{ext}= 100$ mT) denote the equilibrium states used in the calculations of the dispersions illustrated in (**b–e**), respectively. The backward volume mode is highlighted in (**e**) with a thick dashed line.

The spin-wave profiles for the case $N_L = 31$ and $d_L=30$ nm are shown in Figs. 5(i-l). Figure 5(i) shows the low-frequency mode [see cyan circle in Fig. 4(b)], which has a similar magnetization distribution to the one shown in Fig. 5(b). For this reason, the low-frequency bulk modes are quite similar in the cases $N_L = 30$ and $N_L = 31$. This similarity is also observed for the high-frequency bulk modes, as shown in Fig. 5(d) and (l). Concerning the surface modes, it is noted that the surface mode evaluated at $k=-30$ rad/$\mu$m [see cyan star in Fig. 4(b)] is completely similar to the one obtained in Fig. 5(f), while the surface mode calculated for a positive wave vector $k=+30$ rad/$\mu$m [green star in Fig. 4(b)] is similar to the one calculated in Fig. 5(e). Consequently, under an external excitation in the frequency range of these surface modes, the case $N_L = 31$ will exhibit two counterpropagating surface modes (with propagation along $\pm z$), one localized on top and the other at the bottom of the system. Nevertheless, for $N_L = 30$, surface modes will propagate only along $- z$ due to the magnetochiral effects induced by the dipolar interaction and will localize at the top or bottom of the system. The strong localization of in-gap chiral surface modes mediated by the dipolar interaction might suggest the possible topological nature of such modes. Indeed, it has been previously reported in Refs. ^56,57^ that topological spin waves emerge naturally in ferromagnetic and antiferromagnetic materials due to the dipolar interaction. However, the apparent topological properties of nonreciprocal spin-wave modes in SAFs reported here should be formally demonstrated by calculating the corresponding topological invariant or winding number, which is beyond the scope of the present work. Nevertheless, the features presented here raise the possibility of exploring topological signatures of spin waves in SAFs as a future and promissory work.

From Fig. 5, one might be tempted to define the localization properties of surface modes in a general way for both even and odd $N_L$. However, in the case with an even number of FM films, their localization strongly depends on the thickness of the ferromagnetic layers. For an odd number of layers, the localization of SMs remains unchanged as the FM layer thickness increases. Specifically, these modes are always concentrated at the top of the multilayer for negative wave vectors ($k<0$), while for $k>0$, they become more localized at the bottom. In contrast, the behavior for even $N_L$ is more intricate. Depending on the FM layer thickness, the surface modes can either be distributed across both outer layers simultaneously or confined to a single FM film at the top or bottom. This behavior is illustrated in Fig. 6, where, for thin or ultrathin films, the modes appear in both the top and bottom FM layers. However, as the thickness $d_L$ increases, they gradually shift to being localized predominantly at either the top or bottom of the multilayered synthetic antiferromagnetic system. Capturing these localization properties accurately requires considering the variations in magnetization oscillations along the FM thickness. This is inherently accounted for in the dynamic matrix method described in this work, highlighting its crucial role in understanding the complex behavior of SMs in multilayered systems.

An important aspect of the previous results is the need for antiparallel alignment of the ferromagnetic layers, where the dipolar interaction creates chiral surface modes. Unlike natural antiferromagnets, the FM layers in synthetic antiferromagnets can be forced to align parallel by applying a moderate in-plane external field. In this case, the chiral properties of the system are lost due to the lack of symmetry breaking along the thickness, making these nonreciprocal and localization properties reconfigurable. This is shown in Fig. 7(a), where the equilibrium magnetization angles $\varphi _n$ of each FM layer are calculated as a function of the external field for $N_L=8$ and $d_L=5$ nm, with the external field applied along the *z* axis, namely $\varphi _{H_\textrm{ext}}=0$ (see Supplementary Information^43^). As the external field increases, the magnetizations transit from antiparallel to parallel, reaching such a parallel state at a critical field $H_\textrm{c}$. For $H_\textrm{ext}<H_\textrm{c}$, spin flop states are observed, as illustrated in Fig. 7(a). The SW dispersion for equilibrium states P$_1$, P$_2$, P$_3$, and P$_4$ [Figs. 7(b)-(e)] shows that the chiral surface mode becomes reciprocal when the layers align parallel. The surface property also vanishes, and all modes are excited throughout the thickness of the structure when $\varphi _n=0$ (not shown). In Fig. 7(e), most modes have a minimum frequency at zero wave vector, while the mode highlighted by a dashed line in Fig. 7(e) shows a symmetric mode with a minimum at a nonzero wave vector. The trend of such a dashed curve corresponds to the backward volume mode, which is characterized by having negative group velocity at small wave vectors. Because such a mode presents an in-phase magnetization precession along the thickness, it is observed at high frequencies due to the negative interlayer exchange ($J_\textrm{inter}<0$).

From an experimental point of view, the predicted SW phenomena could be measured via time-resolved x-ray tomo-ptychography^58–60^. Here, ptychography would allow for a spatial resolution of the order of 10 nm and probing of thick samples ($\sim 1 \mu$m) in phase contrast. On the other hand, tomography would provide three-dimensional imaging and by that separation of individual ferromagnetic layers of 30 nm thickness and below. Furthermore, distinct magnon surface modes would be readily detectable by local conventional tunneling magnetoresistance sensors or the use of magnetometry based on single nitrogen-vacancy centres in diamond^61^. Spin-wave nonreciprocity paves the way for diverse applications in magnon-based technologies. One of the principal uses is in the design and implementation of devices such as circulators and isolators, which are essential components for controlling signal flow in magnonic circuits^20^. Nonreciprocal spin-wave propagation also enables unidirectional signal transmission, making it possible to create magnonic diodes and other components crucial for next-generation spintronic and magnonic communication systems^30^.

## Conclusions

This research provides significant insights into the complex spin-wave dynamics in multilayered synthetic antiferromagnets, revealing a significant interplay between the number of ferromagnetic layers ($N_L$) and their thickness, which influences the nonreciprocity of spin-wave modes. The clear distinction in dispersion behavior between systems with even and odd $N_L$, where even $N_L$ induces nonreciprocal dispersion due to dynamic dipolar interactions and odd $N_L$ results in a reciprocal behavior, highlights a crucial symmetry aspect of these structures. The emergence and clustering of bulk modes within a narrow frequency range for a given wave vector, as $N_L$ increases, highlights the impact of system thickness on the frequency distribution of bulk modes. The observed chiral properties of surface modes in systems with even $N_L$ add complexity and enhance the functional potential of these multilayer systems. Finally, the revealed tunability of these magnetic properties through external fields, enabling transitions from antiparallel to parallel alignment, demonstrates the reconfigurability and adaptability of synthetic antiferromagnets, paving the way for advanced applications in magnonic devices.

## Supplementary Information

Supplementary Information.

## Data Availability

The data that support the findings of this study are available from the corresponding author upon reasonable request.

## References

[1] Duine, R., Lee, K.-J., Parkin, S. S. & Stiles, M. D. Synthetic antiferromagnetic spintronics. *Nature physics* **14**, 217–219 (2018). 29910827 10.1038/s41567-018-0050-y PMC5997292

[2] Wang, K., Bheemarasetty, V. & Xiao, G. Spin textures in synthetic antiferromagnets: Challenges, opportunities, and future directions. *APL Materials* **11** (2023).

[3] Grünberg, P., Schreiber, R., Pang, Y., Brodsky, M. & Sowers, H. Layered magnetic structures: Evidence for antiferromagnetic coupling of fe layers across cr interlayers. *Physical review letters* **57**, 2442 (1986). 10033726 10.1103/PhysRevLett.57.2442

[4] Bruno, P. & Chappert, C. Ruderman-kittel theory of oscillatory interlayer exchange coupling. *Physical Review B* **46**, 261 (1992). 10.1103/physrevb.46.261 10002208

[5] Fert, A., Grünberg, P., Barthélémy, A., Petroff, F. & Zinn, W. Layered magnetic structures: interlayer exchange coupling and giant magnetoresistance. *Journal of Magnetism and Magnetic Materials* **140**, 1–8 (1995).

[6] Stiles, M. D. Interlayer exchange coupling. *Journal of Magnetism and Magnetic Materials* **200**, 322–337 (1999).

[7] Parkin, S. S. Systematic variation of the strength and oscillation period of indirect magnetic exchange coupling through the 3d, 4d, and 5d transition metals. *Physical Review Letters* **67**, 3598 (1991). 10044776 10.1103/PhysRevLett.67.3598

[8] Yang, Q. et al. Ionic liquid gating control of rkky interaction in fecob/ru/fecob and (pt/co) 2/ru/(co/pt) 2 multilayers. *Nature communications* **9**, 991 (2018). 29515180 10.1038/s41467-018-03356-z PMC5841336

[9] Chongthanaphisut, P. et al. Interlayer exchange coupling in ferromagnetic semiconductor trilayers with out-of-plane magnetic anisotropy. *Scientific reports* **9**, 4740 (2019). 30894576 10.1038/s41598-019-41138-9 PMC6427040

[10] Chen, B. et al. All-oxide-based synthetic antiferromagnets exhibiting layer-resolved magnetization reversal. *Science* **357**, 191–194 (2017). 28706069 10.1126/science.aak9717

[11] Fechner, M., Zahn, P., Ostanin, S., Bibes, M. & Mertig, I. Switching magnetization by 180 with an electric field. *Physical review letters* **108**, 197206 (2012). 23003084 10.1103/PhysRevLett.108.197206

[12] Newhouse-Illige, T. et al. Voltage-controlled interlayer coupling in perpendicularly magnetized magnetic tunnel junctions. *Nature communications* **8**, 15232 (2017). 28508882 10.1038/ncomms15232 PMC5440805

[13] Lonsky, M. & Hoffmann, A. Dynamic fingerprints of synthetic antiferromagnet nanostructures with interfacial dzyaloshinskii–moriya interaction. *Journal of Applied Physics* **132** (2022).

[14] Legrand, W. et al. Room-temperature stabilization of antiferromagnetic skyrmions in synthetic antiferromagnets. *Nature materials* **19**, 34–42 (2020). 31477905 10.1038/s41563-019-0468-3

[15] Yoshida, C. et al. Enhanced thermal stability in perpendicular top-pinned magnetic tunnel junction with synthetic antiferromagnetic free layers. *IEEE transactions on magnetics* **49**, 4363–4366 (2013).

[16] Yang, C.-L. & Lai, C.-H. High thermal durability of ru-based synthetic antiferromagnet by interfacial engineering with re insertion. *Scientific reports* **11**, 15214 (2021). 34312435 10.1038/s41598-021-94640-4 PMC8313549

[17] Gallardo, R. et al. Reconfigurable spin-wave nonreciprocity induced by dipolar interaction in a coupled ferromagnetic bilayer. *Physical Review Applied* **12**, 034012 (2019).

[18] Sluka, V. et al. Emission and propagation of 1d and 2d spin waves with nanoscale wavelengths in anisotropic spin textures. *Nature nanotechnology* **14**, 328–333 (2019). 30804478 10.1038/s41565-019-0383-4

[19] Ishibashi, M. et al. Switchable giant nonreciprocal frequency shift of propagating spin waves in synthetic antiferromagnets. *Science advances* **6**, eaaz6931 (2020). 10.1126/sciadv.aaz6931 PMC7182415 32494648

[20] Barman, A. et al. The 2021 magnonics roadmap. *Journal of Physics: Condensed Matter* **33**, 413001 (2021). 10.1088/1361-648X/abec1a 33662946

[21] Gallardo, R., Alvarado-Seguel, P. & Landeros, P. Unidirectional chiral magnonics in cylindrical synthetic antiferromagnets. *Physical Review Applied* **18**, 054044 (2022).

[22] Gladii, O. et al. Spin-wave nonreciprocity at the spin-flop transition region in synthetic antiferromagnets. *Physical Review B* **107**, 104419 (2023).

[23] Gallardo, R. et al. Coherent magnons with giant nonreciprocity at nanoscale wavelengths. *ACS nano* **18**, 5249–5257 (2024). 38314709 10.1021/acsnano.3c08390 PMC10883124

[24] Girardi, D. et al. Three-dimensional spin-wave dynamics, localization and interference in a synthetic antiferromagnet. *Nature Communications* **15**, 3057 (2024). 38594233 10.1038/s41467-024-47339-9 PMC11004151

[25] Sorokin, S. et al. Magnetization dynamics in synthetic antiferromagnets: Role of dynamical energy and mutual spin pumping. *Physical Review B* **101**, 144410 (2020).

[26] Sud, A. et al. Tunable magnon-magnon coupling in synthetic antiferromagnets. *Physical Review B* **102**, 100403 (2020). 10.1103/PhysRevLett.125.017203 32678634

[27] Li, M., Lu, J. & He, W. Symmetry breaking induced magnon-magnon coupling in synthetic antiferromagnets. *Physical Review B* **103**, 064429 (2021).

[28] Dai, C. & Ma, F. Strong magnon–magnon coupling in synthetic antiferromagnets. *Applied Physics Letters* **118** (2021).

[29] Hu, B. & He, W. Tunable magnon-magnon coupling mediated by in-plane magnetic anisotropy in synthetic antiferromagnets. *Journal of Magnetism and Magnetic Materials* **565**, 170283 (2023).

[30] Wang, Q., Csaba, G., Verba, R., Chumak, A. V. & Pirro, P. Nanoscale magnonic networks. *Physical Review Applied* **21**, 040503 (2024).

[31] Camley, R. E. Long-wavelength surface spin waves on antiferromagnets. *Phys. Rev. Lett.* **45**, 283–286 (1980).

[32] Grünberg, P. & Mika, K. Magnetostatic spin-wave modes of a ferromagnetic multilayer. *Phys. Rev. B* **27**, 2955–2963 (1983). 10.1103/physrevb.31.4465 9936378

[33] Camley, R. E., Rahman, T. S. & Mills, D. L. Magnetic excitations in layered media: Spin waves and the light-scattering spectrum. *Phys. Rev. B* **27**, 261–277 (1983).

[34] Almeida, N. S. & Mills, D. L. Effective-medium theory of long-wavelength spin waves in magnetic superlattices. *Phys. Rev. B* **38**, 6698–6710 (1988). 10.1103/physrevb.38.6698 9945348

[35] Hillebrands, B. Spin-wave calculations for multilayered structures. *Phys. Rev. B* **41**, 530–540 (1990). 10.1103/physrevb.41.530 9992789

[36] Camley, R. E. & Stamps, R. L. *Green’s functions in magnetic multilayered structures*, 237–286.

[37] Stamps, R. L. & Camley, R. E. Spin waves in antiferromagnetic thin films and multilayers: Surface and interface exchange and entire-cell effective-medium theory. *Phys. Rev. B* **54**, 15200–15209 (1996). 10.1103/physrevb.54.15200 9985582

[38] Magnetization dynamics in thin films and multilayers. *Journal of Magnetism and Magnetic Materials* **200**, 583–597 (1999).

[39] Chapter 4 static, dynamic, and thermal properties of magnetic multilayers and nanostructures. In Mills, D. & Bland, J. (eds.) *Nanomagnetism: Ultrathin Films, Multilayers and Nanostructures*, vol. 1 of *Contemporary Concepts of Condensed Matter Science*, 77–114 (Elsevier, 2006).

[40] Wintz, S. et al. Magnetic vortex cores as tunable spin-wave emitters. *Nature Nanotechnology* **11**, 948–953. 10.1038/nnano.2016.117 (2016). 27428277 10.1038/nnano.2016.117

[41] Gallardo, R. et al. Spin-wave non-reciprocity in magnetization-graded ferromagnetic films. *New Journal of Physics* **21**, 033026 (2019).

[42] Gurevich, A. G. & Melkov, G. A. *Magnetization oscillations and waves* (CRC press, 2020).

[43] URL_will_be_inserted_by_publisher.

[44] Jiménez-Bustamante, J. et al. Static and dynamic properties of noncollinear magnetized ferromagnetic films. *Physical Review B* **109**, 094403 (2024).

[45] Körber, L. et al. TetraX: Finite-Element Micromagnetic-Modeling Package, doi:10.14278/rodare.1418 (2022).

[46] Körber, L. et al. Finite-element dynamic-matrix approach for propagating spin waves: Extension to mono-and multi-layers of arbitrary spacing and thickness. *AIP Advances* **12** (2022).

[47] Körber, L., Quasebarth, G., Otto, A. & Kákay, A. Finite-element dynamic-matrix approach for spin-wave dispersions in magnonic waveguides with arbitrary cross section. *AIP Advances* **11** (2021).

[48] Hillebrands, B. *Brillouin light scattering from layered magnetic structures, 174–289* (Springer, Berlin Heidelberg, Berlin, Heidelberg, 2000).

[49] Owerre, S. Strain-induced topological magnon phase transitions: Applications to kagome-lattice ferromagnets. *Journal of Physics: Condensed Matter* **30**, 245803 (2018). 29741490 10.1088/1361-648X/aac365

[50] Fu, Y. & Qin, H. Topological phases and bulk-edge correspondence of magnetized cold plasmas. *Nature Communications* **12**, 3924 (2021). 34168159 10.1038/s41467-021-24189-3 PMC8225675

[51] Barman, A. & Haldar, A. Time-domain study of magnetization dynamics in magnetic thin films and micro-and nanostructures. In *Solid State Physics*, vol. 65, 1–108 (Elsevier, 2014).

[52] Wakatsuki, R., Ezawa, M. & Nagaosa, N. Domain wall of a ferromagnet on a three-dimensional topological insulator. *Scientific reports* **5**, 13638 (2015). 26323943 10.1038/srep13638 PMC4555097

[53] Roldán-Molina, A., Nunez, A. & Fernández-Rossier, J. Topological spin waves in the atomic-scale magnetic skyrmion crystal. *New Journal of Physics* **18**, 045015 (2016).

[54] Malz, D., Knolle, J. & Nunnenkamp, A. *Topological magnon amplification. Nature communications* **10**, 3937 (2019). 31477725 10.1038/s41467-019-11914-2 PMC6718654

[55] Bayer, C. et al. Spin-wave excitations in finite rectangular elements. *Spin dynamics in confined magnetic structures III* 57–103 (2006).

[56] Yamamoto, K. et al. Topological characterization of classical waves: The topological origin of magnetostatic surface spin waves. *Physical review letters* **122**, 217201 (2019). 31283306 10.1103/PhysRevLett.122.217201

[57] Liu, J., Wang, L. & Shen, K. Dipolar spin waves in uniaxial easy-axis antiferromagnets: A natural topological nodal-line semimetal. *Physical Review Research* **2**, 023282 (2020).

[58] Donnelly, C. et al. Three-dimensional magnetization structures revealed with x-ray vector nanotomography. *Nature* **547**, 328–331 (2017). 28726832 10.1038/nature23006

[59] Neethirajan, J. et al. Soft x-ray phase nanomicroscopy of micrometer-thick magnets. *Physical Review X* **14**, 031028 (2024).

[60] Butcher, T. A., Phillips, N. W., Levitan, A. L., Raabe, J. & Finizio, S. Ptychographic imaging of magnetic domain wall dynamics. *arXiv preprint* arXiv:2408.14172 (2024).

[61] Van der Sar, T., Casola, F., Walsworth, R. & Yacoby, A. Nanometre-scale probing of spin waves using single electron spins. *Nature communications* **6**, 7886 (2015). 26249673 10.1038/ncomms8886 PMC4918315

