# Supplementary material for: Impact of layer count and thickness on spin wave modes in multilayer synthetic antiferromagnets
Source: Sci Rep. 2025 Jul 1;15:21545. doi: 10.1038/s41598-025-08393-5 (PMC12219261; doi:10.1038/s41598-025-08393-5)
Supplement: Supplementary file 1 — Supplementary Information. [file 41598_2025_8393_MOESM1_ESM.pdf]

SUPPLEMENTARY INFORMATION: Impact of layer count and thickness on spin wave modes in multilayer synthetic antiferromagnets

J. Jiménez-Bustamante,[1] N. Vidal-Silva,[2] A. Kákay,[3] S. Wintz,[4] and R. A. Gallardo[1,*]

[1]*Departamento de Física, Universidad Técnica Federico Santa María, Avenida España 1680, Valparaíso, Chile*
[2]*Departamento de Ciencias Físicas, Universidad de La Frontera, Casilla 54-D, Temuco, Chile*
[3]*Helmholtz-Zentrum Dresden-Rossendorf, Institute of Ion Beam Physics and Materials Research, Bautzner Landstraße 400, 01328 Dresden, Germany*
[4]*Helmholtz-Zentrum Berlin, 12489 Berlin, Germany*

I. EFFECTIVE FIELDS

A. Zeeman and Intralayer exchange fields

For the in-plane case, the external field is given by

$$\mathbf{H}_{\rm ext} = H_{\rm ext}\left[\cos(\varphi_H - \varphi_n)\hat{Z}_n + \sin(\varphi_H - \varphi_n)\hat{X}_n\right].$$

Angle $\varphi_{H_{\rm ext}}$ is defined from the z axis in such a way that for $\varphi_{H_{\rm ext}} = 0$, the external field is pointing along z, while for $\varphi_{H_{\rm ext}} = \pi/2$ the field is along x axis.

Intralayer exchange field is obtained from $\mathbf{H}^{{\rm ex}(n)} = \left(\nabla\cdot[\ell_{\rm ex}]^2\nabla\right)\mathbf{M}_n$, where $\ell_{\rm ex} = \sqrt{2A_{\rm ex}/\mu_0(M_{\rm s_n})^2}$ is the exchange length. Therefore, writing the magnetization in the linear regime and taking into account that the exchange length is constant, the exchange field components are $H^{\rm ex0}_{Z_n} = H^{\rm ex0}_{X_n} = 0$ and $h^{\rm ex}_{X_n,Y_n} = -\ell^2_{\rm ex}k^2 m_{X_n,Y_n}$.

B. Dipolar fields

The dynamic dipolar field is produced by volumetric and surface bounded magnetic charges. For the volumetric ones, they are given by $\rho = -\nabla_{\mathbf{r}}\cdot\mathbf{M}_n = -dM_{z_n}/dz$, where it has been a uniform distribution of the dynamic magnetization along the thickness of each n-th sublayer. Then, by considering $m_{X_n,Y_n}(\mathbf{r}) = m_{X_n,Y_n}e^{i\mathbf{k}\cdot\mathbf{r}}$ with $\mathbf{k}\cdot\mathbf{r} = kz$, the volumetric magnetic charge is $\rho = im_{X_n}k\sin\varphi_n e^{i\mathbf{k}\cdot\mathbf{r}}$. The magnetic potential is calculated from

$$\phi_V(y) = \frac{1}{4\pi}im_{X_n}k\sin\varphi_n\int\frac{e^{i\mathbf{k}\cdot\mathbf{r}'}}{|\mathbf{r}-\mathbf{r}'|}\mathrm{d}^3\mathbf{r}'. \tag{S.1}$$

The integral in Eq. (S.1) can be analytically evaluated in the in-plane coordinates, so that

$$\int\frac{e^{i\mathbf{k}\cdot\mathbf{r}'}}{|\mathbf{r}-\mathbf{r}'|}\mathrm{d}^3\mathbf{r}' \;=\; 2\pi e^{i\mathbf{k}\cdot\mathbf{r}}I_n(y), \tag{S.2}$$

where

$$I_n(y) = \int_{\xi_n}^{\xi_n+d_n}\frac{e^{-|\mathbf{k}||y-y'|}}{|\mathbf{k}|}\mathrm{d}y'. \tag{S.3}$$

where $\xi_n = (n-1)(d_n+s)$. Now, it can be seen that the solution of the integral $I_n(y)$ requires addressing the conditions $y' > y$ and $y' < y$. If the potential will be evaluated in the same sublayer

n, the integral is

$$I_n(y) = \int_{\xi_n}^{y} \frac{e^{-|\mathbf{k}|(y-y')}}{|\mathbf{k}|} \mathrm{d}y' + \int_{y}^{d+\xi_n} \frac{e^{-|\mathbf{k}|(y'-y)}}{|\mathbf{k}|} \mathrm{d}y'$$
$$= 2\frac{1-\cosh[|\mathbf{k}|\,(d_n/2+\xi_n-y)]e^{-|\mathbf{k}|d_n/2}}{|\mathbf{k}|^2}. \quad \text{(S.4)}$$

Then, the η-component of the dipolar field can be calculated from $h_\eta^V(y) = -\partial_\eta \phi_V(y)$. Of course, such a field will depend on the normal coordinate y. Nevertheless, at this point, it is assumed that the variation of this field is negligible so that the average over the sublayer thickness is calculated, namely $\langle h_\nu^V \rangle_n = 1/d_n \int_{\xi_n}^{\xi_n+d_n} h_\nu^V(y)dy$. Therefore, $\langle h_y^V \rangle_n = 0$ and

$$\langle h_z^V \rangle_n = m_{X_n} [1-\zeta(k,d_n)] \sin\varphi_n e^{ikz}, \quad \text{(S.5)}$$

where

$$\zeta(k,d_n) = \frac{2\sinh[|k|\,d_n/2]\,e^{-|k|d_n/2}}{d_n\,|k|}. \quad \text{(S.6)}$$

Finally, the projection to the local reference system gives $\langle h_{X_n}^V \rangle = -\langle h_z^V \rangle \sin\varphi_n$, this is

$$\langle h_{X_n}^V \rangle_n = -m_{X_n} [1-\zeta(k,d_n)] \sin^2\varphi_n e^{ikz}. \quad \text{(S.7)}$$

Now, the magnetic potential (generated by a sublayer n) is evaluated in an upper sublayer ν, so that $y-y' > 0$. Therefore,

$$I_n(y) = \Psi_k \frac{e^{|k|(\xi_n - y)}}{|k|^2} \quad \text{(S.8)}$$

with

$$\Psi_k = (e^{d_n|k|}-1). \quad \text{(S.9)}$$

By calculating the field and its average over the ν-th sublayer, the dipolar field components are

$$\langle h_{X_\nu}^V \rangle_{\nu>n} = -m_{X_n} \sin\varphi_n \sin\varphi_\nu \frac{\Psi_k^2 e^{|k|(\xi_n-\xi_\nu-d_n)}}{2d_\nu|k|} e^{ikz} \quad \text{(S.10)}$$

and

$$\langle h_{Y_\nu}^V \rangle_{\nu>n} = i m_{X_n} k \sin\varphi_n \frac{\Psi_k^2 e^{|k|(\xi_n-\xi_\nu-d_n)}}{2d_\nu|k|^2} e^{ikz}. \quad \text{(S.11)}$$

Note that the previous case considers $\nu > n$ since the sublayer ν is located above sublayer n. Now, for the case $\nu < n$, it is straightforward to show that

$$\left\langle h^V_{X_\nu} \right\rangle_{\nu<n} = -m_{X_n} \sin\varphi_n \sin\varphi_\nu \frac{\Psi^2_k e^{|\mathbf{k}|(\xi_\nu-\xi_n-d_n)}}{2d_\nu |k|} e^{ikz} \tag{S.12}$$

and

$$\left\langle h^V_{Y_\nu} \right\rangle_{\nu<n} = -im_{X_n} k \sin\varphi_n \frac{\Psi^2_k e^{|k|(\xi_\nu-\xi_n-d_n)}}{2d_\nu |k|^2} e^{ikz}. \tag{S.13}$$

The magnetic potential generated by surface magnetic charges is given by

$$\phi_S(y) = \frac{1}{4\pi} \int \frac{M_n(\mathbf{r}')}{|\mathbf{r}-\mathbf{r}'|} \mathrm{d}^2 S', \tag{S.14}$$

where $M_n(\mathbf{r}')$ is the normal component of the magnetization. In this case, due to the extended geometry of the layer, only the m_{Y_n} dynamic magnetization component contributes to such potential. Thus,

$$\begin{aligned} \phi_S &= \frac{1}{4\pi} \int \frac{m_{Y_n} e^{i\mathbf{k}\cdot\mathbf{r}'}}{|\mathbf{r}-\mathbf{r}'|} \mathrm{d}^2 S'_{y'=d+\xi_n} \\ &\quad -\frac{1}{4\pi} \int \frac{m_{Y_n} e^{i\mathbf{k}\cdot\mathbf{r}'}}{|\mathbf{r}-\mathbf{r}'|} \mathrm{d}^2 S'_{y'=\xi_n}. \end{aligned} \tag{S.15}$$

Then, by following the considerations used for the contribution of the volumetric charges, the field due to surface bound charges are $\left\langle h^S_{X_n} \right\rangle_n = 0$,

$$\left\langle h^S_{Y_n} \right\rangle_n = -m_{Y_n} \zeta(k, d_n) e^{ikz}, \tag{S.16}$$

$$\left\langle h^S_{X_\nu} \right\rangle_{\nu>n} = im_{Y_n} k \sin\varphi_\nu \frac{\Psi^2_k e^{|k|(\xi_n-\xi_\nu-d_n)}}{2d_\nu |k|^2} e^{ikz}, \tag{S.17}$$

$$\left\langle h^S_{Y_\nu} \right\rangle_{\nu>n} = m_{Y_n} \frac{\Psi^2_k e^{|k|(\xi_n-\xi_\nu-d_n)}}{2d_\nu |k|} e^{ikz}, \tag{S.18}$$

$$\left\langle h^S_{X_\nu} \right\rangle_{\nu<n} = -im_{Y_n} k \sin\varphi_\nu \frac{\Psi^2_k e^{|k|(\xi_\nu-\xi_n-d_n)}}{2d_\nu |k|^2} e^{ikz} \tag{S.19}$$

and

$$\left\langle h^S_{Y_\nu} \right\rangle_{\nu<n} = m_{Y_n} \frac{\Psi^2_k e^{|k|(\xi_\nu-\xi_n-d_n)}}{2d_\nu |k|} e^{ikz}. \tag{S.20}$$

C. Interlayer exchange field

The interlayer exchange energy, per unit area, is given by

$$\varepsilon^{(n)}_{\mathrm{Int}} = -\sum_\nu J_{n\nu} \frac{\mathbf{M}_n \cdot \mathbf{M}_\nu}{M_{\mathrm{s}_n} M_{\mathrm{s}_\nu}}. \tag{S.21}$$

Then, the dynamic effective exchange field components are

$$h^{\mathrm{Int}}_{Y_n} = \sum_{\nu} \left(\delta^{\nu}_{n+1} + \delta^{\nu}_{n-1}\right) \frac{J_{n\nu}}{d_n \mu_0 M_{\mathrm{s}_n} M_{\mathrm{s}_\nu}} m_{Y_\nu} \tag{S.22}$$

and

$$h^{\mathrm{Int}}_{X_n} = \sum_{\nu} \left(\delta^{\nu}_{n+1} + \delta^{\nu}_{n-1}\right) \frac{J_{n\nu}}{d_n \mu_0 M_{\mathrm{s}_n} M_{\mathrm{s}_\nu}} \cos(\varphi_n - \varphi_\nu) m_{X_\nu}. \tag{S.23}$$

The static components are

$$H^{\mathrm{Int}}_{Z_n} = \sum_{\nu} \left(\delta^{\nu}_{n+1} + \delta^{\nu}_{n-1}\right) \frac{J_{n\nu}}{d_n \mu_0 M_{\mathrm{s}_n}} \cos(\varphi_n - \varphi_\nu) \tag{S.24}$$

and

$$H^{\mathrm{Int}}_{X_n} = \sum_{\nu} \left(\delta^{\nu}_{n+1} + \delta^{\nu}_{n-1}\right) \frac{J_{n\nu}}{d \mu_0 M_{\mathrm{s}_\nu}} \sin(\varphi_n - \varphi_\nu). \tag{S.25}$$

Note that the Kronecker symbol δ^i_i (with $\delta^i_i = 1$ if $i = j$ and zero otherwise) is introduced to account only an exchange interaction between nearest neighbours. Besides, if n and ν correspond to the same ferromagnetic layer, the exchange constant becomes $J_{n\nu} = J_{\mathrm{intra}} = 2A_{\mathrm{ex}}/d$, where such a constant represents an intralayer exchange coupling. In the case that n and ν correspond to the sublayers separated by the spacer s, then $J_{n\nu} = J_{\mathrm{inter}}$, where $J_{\mathrm{inter}} < 0$ is the interlayer exchange constant that couple different ferromagnetic layers.

II. MATRIX ELEMENTS

Overall, the dynamic effective field components contain the interaction between different layers. Therefore, generalizing, these fields can be expressed as

$$M_{\mathrm{s}_n} h^{\mathrm{e}}_{X_n} = \sum_{\nu} T^{X_\nu}_{X_n} m_{X_\nu} + \sum_{\nu} T^{Y_\nu}_{X_n} m_{Y_\nu}, \tag{S.26}$$

and

$$M_{\mathrm{s}_n} h^{\mathrm{e}}_{Y_n} = \sum_{\nu} T^{X_\nu}_{Y_n} m_{X_\nu} + \sum_{\nu} T^{Y_\nu}_{Y_n} m_{Y_\nu}. \tag{S.27}$$

Therefore, the equations of motion for each magnetization component become

$$\begin{aligned} i\frac{\omega}{\gamma\mu_0} m_{X_n} &= m_{Y_n} H^{\mathrm{e0}}_{Z_n} - \sum_{\nu} T^{X_\nu}_{Y_n} m_{X_\nu} - \sum_{\nu} T^{Y_\nu}_{Y_n} m_{Y_\nu} \\ i\frac{\omega}{\gamma\mu_0} m_{Y_n} &= -m_{X_n} H^{\mathrm{e0}}_{Z_n} + \sum_{\nu} T^{X_\nu}_{X_n} m_{X_\nu} + \sum_{\nu} T^{Y_\nu}_{X_n} m_{Y_\nu}. \end{aligned}$$

Thus, as mentioned before, the previous set of equations can be cast in a matrix form $i\omega\mathbf{m}_n = \gamma\mu_0\tilde{\mathbf{A}}\mathbf{m}_n$, where

$$\tilde{\mathbf{A}} = \begin{pmatrix} A_{X_n}^{X_\nu} & A_{X_n}^{Y_\nu} \\ A_{Y_n}^{X_\nu} & A_{Y_n}^{Y_\nu} \end{pmatrix}.$$

Besides, the matrix elements can be separated in terms of intralayer nature, namely $n = \nu$,

$$A_{X_n}^{X_n} = -T_{Y_n}^{X_n}\ ; \quad A_{X_n}^{Y_n} = -T_{Y_n}^{Y_n} + H_{Z_n}^{\mathrm{e}0} \tag{S.29}$$

$$A_{Y_n}^{X_n} = -H_{Z_n}^{\mathrm{e}0} + T_{X_n}^{X_n}\ ; \quad A_{Y_n}^{Y_n} = T_{X_n}^{Y_n}, \tag{S.30}$$

and terms that contain interlayer energies ($n \neq \nu$), this is

$$A_{X_n}^{X_\nu} = -T_{Y_n}^{X_\nu}\ ; \quad A_{X_n}^{Y_\nu} = -T_{Y_n}^{Y_\nu} \tag{S.31}$$

$$A_{Y_n}^{X_\nu} = T_{X_n}^{X_\nu}\ ; \quad A_{Y_n}^{Y_\nu} = T_{X_n}^{Y_\nu}. \tag{S.32}$$

Note that the terms $T_{\xi_n}^{\xi'_\nu}$ are obtained from the calculation of the dynamic effective fields. According to the effective field given in Appendix I, the matrix terms for $n = \nu$ are $T_{Y_n}^{X_n} = T_{X_n}^{Y_n} = 0$,

$$T_{X_n}^{X_n} = -M_{\mathrm{s}_n}\left[1 - \zeta(k, d_n)\right]\sin^2\varphi_n - M_{\mathrm{s}_n}\ell_{\mathrm{ex}}^2 k^2 \tag{S.33}$$

and

$$T_{Y_n}^{Y_n} = -M_{\mathrm{s}_n}\zeta(k, d_n) - M_{\mathrm{s}_n}\ell_{\mathrm{ex}}^2 k^2. \tag{S.34}$$

On the other hand, the interlayer terms are given by

$$T_{Y_n}^{X_\nu} = \mathrm{sgn}(\nu - n)ikM_{\mathrm{s}_n}\sin\varphi_\nu \frac{\Psi_k^2 e^{-|k|(|\xi_\nu - \xi_n| + d_\nu)}}{2d_n|k|^2}, \tag{S.35}$$

$$T_{X_n}^{Y_\nu} = \mathrm{sgn}(\nu - n)ikM_{\mathrm{s}_n}\sin\varphi_n \frac{\Psi_k^2 e^{-|k|(|\xi_\nu - \xi_n| + d_\nu)}}{2d_n|k|^2}, \tag{S.36}$$

$$T_{X_n}^{X_\nu} = \sum_\nu \frac{J_{n\nu}\cos(\varphi_n - \varphi_\nu)}{d_n\mu_0 M_{\mathrm{s}_\nu}}\left(\delta_{n+1}^\nu + \delta_{n-1}^\nu\right) - M_{\mathrm{s}_n}\sin\varphi_n\sin\varphi_\nu \frac{\Psi_k^2 e^{-|k|(|\xi_\nu - \xi_n| + d_\nu)}}{2d_n|k|}, \tag{S.37}$$

and

$$T_{Y_n}^{Y_\nu} = \sum_\nu \frac{J_{n\nu}}{d_n\mu_0 M_{\mathrm{s}_\nu}}\left(\delta_{n+1}^\nu + \delta_{n-1}^\nu\right) + M_{\mathrm{s}_\nu}\frac{\Psi_k^2 e^{-|k|(|\xi_\nu - \xi_n| + d_\nu)}}{2d_n|k|}. \tag{S.38}$$

Finally, the static effective field components of the fields are

$$H_{Z_\nu}^0 = H\cos(\varphi_H - \varphi_\nu) + \sum_\nu \frac{J_{n\nu}\cos(\varphi_n - \varphi_\nu)}{d_n\mu_0 M_{\mathrm{s}_n}}\left(\delta_{n+1}^\nu + \delta_{n-1}^\nu\right) \tag{S.39}$$

and

$$H_{X_\nu}^0 = H\sin(\varphi_H - \varphi_\nu) + \sum_\nu \frac{J_{n\nu}\sin(\varphi_n - \varphi_\nu)}{d_n\mu_0 M_{\mathrm{s}_n}}\left(\delta_{n+1}^\nu + \delta_{n-1}^\nu\right). \tag{S.40}$$

Note that the last term is null ($H_{X_\nu}^{\mathrm{e}0} = 0$) according to the equilibrium conditions of the system.
